# A Novel Signature Integrated of Immunoglobulin, Glycosylation and Anti-Viral Genes to Predict Prognosis for Breast Cancer

**DOI:** 10.3389/fgene.2022.834731

**Published:** 2022-04-01

**Authors:** Shengshan Xu, Yuchen Liu, Hansu Ma, Shuo Fang, Shoupeng Wei, Xiaoping Li, Zhuming Lu, Youbin Zheng, Tong Liu, Xiaojian Zhu, Dongming Xu, Yihang Pan

**Affiliations:** ^1^ Department of Thoracic Surgery, Affiliated Jiangmen Hospital of Sun Yat-sen University, Jiangmen, China; ^2^ Scientific Research Center, The Seventh Affiliated Hospital, Sun Yat-sen University, Shenzhen, China; ^3^ Department of Oncology, The Seventh Affiliated Hospital, Sun Yat-Sen University, Shenzhen, China; ^4^ Department of Breast, Affiliated Jiangmen Hospital of Sun Yat-sen University, Jiangmen, China; ^5^ Department of Radiology, Jiangmen Wuyi Hospital of Traditional Chinese Medicine, Jiangmen, China; ^6^ Department of Thoracic Surgery, First Hospital of Jilin University, Changchun, China; ^7^ Department of Neurosurgery, The Country Hospital of Qianguo, Songyuan, China

**Keywords:** breast cancer, gene signature, prognosis mode, immunotharapy, glycosylation

## Abstract

**Background:** Aberrant glycosylation is significantly related to the occurrence, progression, metastasis, and drug resistance of tumors. It is essential to identify glycosylation and related genes with prognostic value for breast cancer.

**Objective:** We aimed to construct and validate a prognostic model based on glycosylation and related genes, and further investigate its prognosis values in validation set and external independent cohorts.

**Materials and Methods:** The transcriptome and clinical data of breast cancer patients were downloaded from The Cancer Genome Atlas (TCGA, *n* = 1072), Molecular Taxonomy of Breast Cancer International Consortium (METABRIC, *n* = 1451), and GSE2741 (*n* = 120). Glycosylation-related genes were downloaded from the Genecards website. Differentially expressed glycosylation-related geneswere identified by comparing the tumor tissues with the adjacent tissues. The TCGA data were randomly divided into training set and validation set in a 1:1 ratio for further analysis. The glycosylation risk-scoring prognosis model was constructed by univariate and multivariate Cox regression analysis, followed by confirmation in TCGA validation, METABRIC, and GEO datasets. Gene set enrichment analysis (GSEA) and Gene ontology analysis for identifying the affected pathways in the high- and low-risk groups were performed.

**Results:** We attained 1072 breast cancer samples from the TCGA database and 786 glycosylation genes from the Genecards website. A signature contains immunoglobulin, glycosylation and anti-viral related genes was constructed to separate BRCA patients into two risk groups. Low-risk patients had better overall survival than high-risk patients (*p* < 0.001). A nomogram was constructed with risk scores and clinical characteristics. The area under time-dependent ROC curve reached 0.764 at 1 year, 0.744 at 3 years, and 0.765 at 5 years in the training set. Subgroup analysis showed differences in OS between the high- and low-risk patients in different subgroups. Moreover, the risk score was confirmed as an independent prognostic indicator of BRCA patients and was potentially correlated with immunotherapy response and drug sensitivity.

**Conclusion:** We identified a novel signature integrated of immunoglobulin (IGHA2), glycosylation-related (SLC35A2) and anti-viral gene (BST2) that was an independent prognostic indicator for BRCA patients. The risk-scoring model could be used for predicting prognosis and immunotherapy in BRCA, thus providing a powerful instrument for combating BRCA.

## Introduction

Globally, breast cancer is the most common cancer among women, and is the leading cause of cancer deaths among women aged 20 to 59 (R. L. Siegel et al., 2021). The cumulative risk of breast cancer is about 5% in women, and the risk of death is 1.4% (E. Hadadi et al., 2020). In recent years, the incidence of breast cancer has continued to increase by about 0.5% annually (R. L. Siegel et al., 2021), which seriously affects women’s health and quality of life. Breast cancer is a highly heterogeneous disease. The main treatments for breast cancer include systemic therapy (chemotherapy, endocrine therapy, targeted therapy, and immunotherapy) and local treatment (surgery and radiotherapy). Breast cancer molecular subtypes were essential indicators for treatment and prognosis. Currently, the majority of patients diagnosed with a specific breast cancer subtype receive the same treatment, even though it has been repeatedly proven that they should adopt differential strategies (S. A. Eccles et al., 2013; S. Fallahpour et al., 2017; W. J. Gradishar et al., 2020). Triple-negative breast cancer (TNBC) is a type of breast cancer that lacks expression of human epidermal growth factor receptor 2 (HER2), progesterone receptor, and estrogen receptor (G. Bianchini et al., 2016). Typically, the prognosis for women with TNBC following metastatic recurrence is much poorer than other subtypes (M. Smid et al., 2008). It is a heterogeneous disease representing about 15% of total breast cancer incidents, which is difficult to treat as lack of available targeted therapies. Chemotherapy remains to be the preferred systemic treatment for TNBC (D. P. Silver et al., 2010), and particularly in those carrying BRCA1 mutations (J. Collignon et al., 2016). This demonstrates the reliable predictive biomarkers are necessary for precise diagnosis and individualized treatment for breast cancer patients, and the precision medicine progress have been fueled by the continuous development of new sequencing and computational technologies.

Genetic and epigenetic alterations are considered the primary causes of cancer development, and the downstream phenotypic changes at the protein level are amongst the driving forces (A. Peixoto et al., 2019). Glycosylation is the most common and complicated post-translational modification for membrane-bound proteins. More than 50–70% of proteins in the circulation are glycosylated, which play important roles in various cellular activities, such as cell growth, differentiation, transformation, and adhesion (Ohtsubo and Marth, 2006). Aberrant glycosylation has been identified as a hallmark of cancer and intimately correlated with cancer occurrence, progression, metastasis, tumor recurrence, and drug resistance (Pinho and Reis, 2015; Rao et al., 2017; A. Chakraborty et al., 2018; Cui et al., 2018). There was also a correlation between glycosylation and antitumor immunity. For example, Freire et al. demonstrated that Tn glycosylation of the MUC6 protein strongly affects the immunogenicity of its B and T cell, and might enable immune escape of tumor cells (T. Freire et al., 2011). Bone marrow stromal antigen 2 (*BST2*) was a type II transmembrane protein, also known as tethered protein, HM1.24 orCD317. BST2 homodimer promotes cancer cell adhesion and enhances cancer cell survival and growth by enhancing proteasomal degradation of pro-apoptotic proteins (Mahauad-Fernandez and Okeoma, 2017). Sayeed et al. indicated that aberrant BST2 overexpression promoted the disappearance of TGFβ-mediated tumor-suppressive effects in breast cancer as a consequence and the ensuing loss of the differentiation program (A. Sayeed et al., 2013). Mahauad Fernandez and Okeoma suggested that BST-2 targets breast cancer cells that are resistant to anoikis via the GRB2/ERK/BIM/Cas3 pathway. Almost all breast tumors express *BST2* to a certain level, and the high expression level of *BST2* was related to progressive malignancies (W. D. Mahauad-Fernandez et al., 2014). Several studies have shown that BST2 regulates the occurrence of gastric cancer, oral squamous cell carcinoma, lung cancer and is involved in tumor metastasis and invasion (W. Wang et al., 2009a; K. H. Fang et al., 2014; W. Liu et al., 2018; W. Liu et al., 2020). Immunoglobulin heavy constant alpha 2 (*IGHA2*), located on chromosome 14, expressed in breast cancer cells and upregulated in advanced breast tumor tissues by comparison with early tumors, was involved in the early stage of the tumor microenvironment remodeling and has been identified as a marker of regional metastasis in lymph nodes. Suki Kang et al. reported that IGHA2 might protect the cells against physiological stresses during the neoplastic process and promote tumor growth in the advanced stages of cancer (S. Kang et al., 2012). Exploring the role of glycosylation, immunoglobulin, and anti-viral in BRCA and related molecules will help us investigate strategies to combat for BRCA. Currently, there is still no study exploring the prognosis value of glycosylation, immunoglobulin, and anti-viral -related genes. Therefore, this study aims to establish a prognostic model for breast cancer based on glycosylation-related genes and evaluate it from multiple dimensions.

## Materials and Methods

### Data Source and Processing

Breast cancer datasets were downloaded from the Molecular Taxonomy of Breast Cancer International Consortium (METABRIC, http://www.cbioportal.org), The Cancer Genome Atlas (TCGA, https://www.cancer.gov), and the Gene Expression Omnibus (GEO, http://www.ncbi.nlm.nih.gov/geo). Patients who met the following selection criteria were included: 1) histologically diagnosed with malignant breast cancer; 2) available RNA expression data; and 3) available OS data. After screening, this study included 1,451 patients from METABRIC, 1,072 patients from TCGA, and 120 patients from GSE2741. Glycosylation-related genes were downloaded from the website of Genecards (https://www.genecards.org). A total of 786 glycosylation genes were analyzed by comparing the tumor tissues with the adjacent tissues to obtain differentially expressed glycosylation-related genes (DEGRGs). A *p* value less than 0.05 was considered statistically significant.

### Construction and Validation of the Risk-Scoring Model

The TCGA BRCA data were randomly assigned into training set and validation set according to the ratio of 1:1. In order to determine the survival-related glycosylation genes, we performed univariate Cox regression, in which *p* < 0.05 was set as a cut-off criterion. Subsequently, multivariate Cox regression was performed to construct a prognostic risk-scoring model, in which the risk score for each patient was calculated according to the following formula:

RiskScore=[(0.9138×Expression value of SLC35A2)+[(−0.2483)×Expression value ofBST2]+[(−0.1002)×Expression value of IGHA2]]

All BRCA patients were assigned to the high- and low-risk groups according to the median risk score in the training set. The difference in OS between these two groups was investigated by the log-rank test and Kaplan-Meier survival analysis. In addition, the distributions of survival status, OS, and risk score in the training set were also plotted.

### Independent Prognostic Analysis

To evaluate the relationship between clinicopathological factors and risk scores on survival time, we used the “Survival” R package to perform univariate and multivariate Cox regression. The time-dependent receiver operator characteristic (ROC) curve was drawn, and the R package “timeROC” was applied to determine the prognostic performance of either clinicopathological factors or risk scores on survival time. *p* < 0.05 was considered statistically significant.

### Subgroup Analysis

We further evaluated the model’s predictive ability by stratifying patients into various subgroups. These variables include age (≤65 and >65 years), tumor stage (I-II and III-IV), T stage (T1-2 and T3-4), N stage (N0 and N1-3), M stage (M0 and M1), estrogen receptor (ER) state (positive and negative), human epidermal growth factor receptor (HER-2) state (positive and negative), triple-negative breast cancer (yes and no). To further confirm the value of the risk score in TNBC and HER-2 positive subgroups, the TNBC and HER-2 positive samples from METABRIC database were used for re-verification. Through clinical survival analysis, the predictive ability of the risk-scoring model in various clinical subgroups was clarified. *p* < 0.05 was considered statistically significant.

### Exploration of the Value of the Risk-Scoring Model in Clinical Utility

To further improve the practical value of the risk-scoring model, a nomogram was constructed by integrating age, tumor stage, T stage, N stage, M stage, and risk score to predict the OS of patients at 1, 3, and 5 years. In addition, the C-index was used to measure the accuracy of the nomogram, and the calibration curve was drawn to evaluate the calibration of the model. The ROC curves of various clinical characteristics were drawn, and the AUC was calculated to judge the performance of the prognosis model. In addition, decision curve analysis (DCA) was used to estimate the maximum clinical benefit by logistic regression analysis.

### Functional Enrichment Analysis

To explore the difference molecular pathways underlying survival prognosis between the high- and low-risk groups, we used the **“clusterProfiler” R package** to perform Gene Ontology (GO) enrichment analysis and Kyoto Encyclopedia of Genes and Genomes (KEGG) pathway analysis. Bubble Diagram visualized the most important pathways in KEGG and each GO category. Gene Enrichment Score Analysis (GSEA) was also applied to determine signaling pathways regulated in patients of the high- and low-risk groups. *p* < 0.05 and FC > 1.2 (or FC < 0.83) were set as the cut-off values.

### Analysis of Tumor Immune Microenvironment

The tumor microenvironment score of each single BRCA patient was estimated using the ESTIMATE algorithm. CIBERSORT algorithm was utilized to evaluate the proportion of 22 human immune cell subsets in the high- and low-risk groups. The “GSVA” R package was applied to perform single-sample gene set enrichment analysis (ssGSEA) to quantify the GSVA scores of the 13 immune signatures. The difference between the expression levels of immune checkpoints in the high- and low-risk groups was further evaluated. *p*-value < 0.05 was considered statistically significant.

### Tumor Mutation Burden Analysis

After obtaining the somatic mutation data in the TCGA BRCA dataset, the “maftools” R package was applied to analyze the tumor mutation burden (TMB) of the training set, TMB value was calculated, and a waterfall chart was drawn. Then it would be assessed whether the TMB scores were related to the risk scores and patient survival probability. We found the median value of TMB and divided TCGA BRCA dataset into high TMB group and low TMB group. Patients were stratified into the new groups by integrating TMB and risk scores, and Kaplan-Meier survival analysis was used. The “Survival” and the “survminer” R packages were used for joint survival analysis. The CBIOPORTAL database (https://www.cbioportal.org/) was used to analyze the mutations profiles in the high-and low-risk groups. Finally, the protein domains where the mutations were located were clarified. *p* < 0.05 was considered statistically significant.

### Relationship Between Risk Scores and Immunotherapy

IMvigor210, a phase II trial of atezolizumab (MPDL3280A) in platinum-treated locally advanced or metastatic urothelial carcinoma. It was downloaded to assess the correlation between risk scores and immunotherapy response. The tumor immune dysfunction and exclusion (TIDE) score was calculated online (http://tide.dfci. harvard. edu/) to assess the immune checkpoint inhibitor response between the high- and low-risk groups (Z. Lin et al., 2021). We downloaded RNA-seq and compound activity: DTP NCI-60 through the CellMiner database (https://discover.nci.nih.gov/cellminer) and excluded FDA status as empty or clinical trial data to explore the relationship between the expression of genes and drug sensitivity. The three-dimensional structures of drugs were obtained through the PubChem database (https://pubchem.ncbi.nlm.nih.gov). *p* < 0.05 was considered statistically significant.

## Results

### Identification of Differential Expression of Glycosylation-Related Genes in Breast Cancer

**FIGURE 1:**
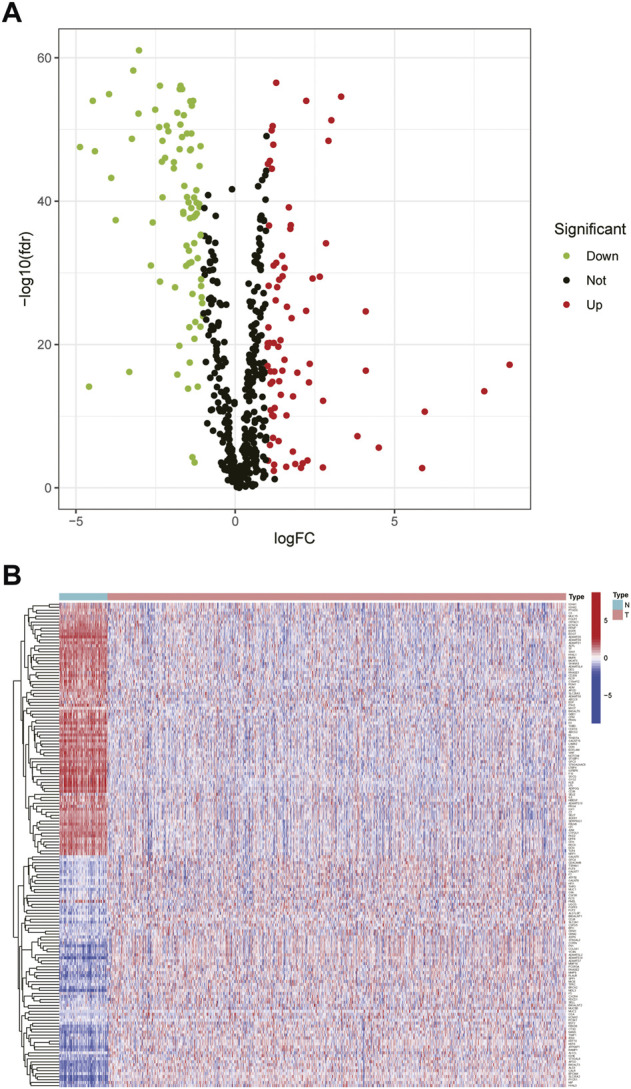
Differential expression of glycosylation-related genes **(A)** The volcano plot showed the up-regulated and down-regulated differential genes in breast tumor tissues compared with adjacent tissues (t-test, Adjust. *p* < 0.05) **(B)** The heat map showed that 163 differential glycosylation-related genes were expressed in tumor tissues and adjacent tissues.

We compiled the gene expression data of breast cancer from the TCGA database and finally got 1,072 tumor tissues and 99 adjacent tissue samples. Around 786 glycosylation-related genes were obtained by the Genecards website with a correlation >2.0 as the cut-off value. Based on standard cut-off values for fold-change in gene expression (|log(FC)|> 1) and false discovery rate (FDR <0.05), the breast cancer tissues had 163 DEGRGs, with 86 down-regulated genes and 77 up-regulated genes (Figures 1A,B).

### Construction of a Risk-Scoring Model

**FIGURE 2:**
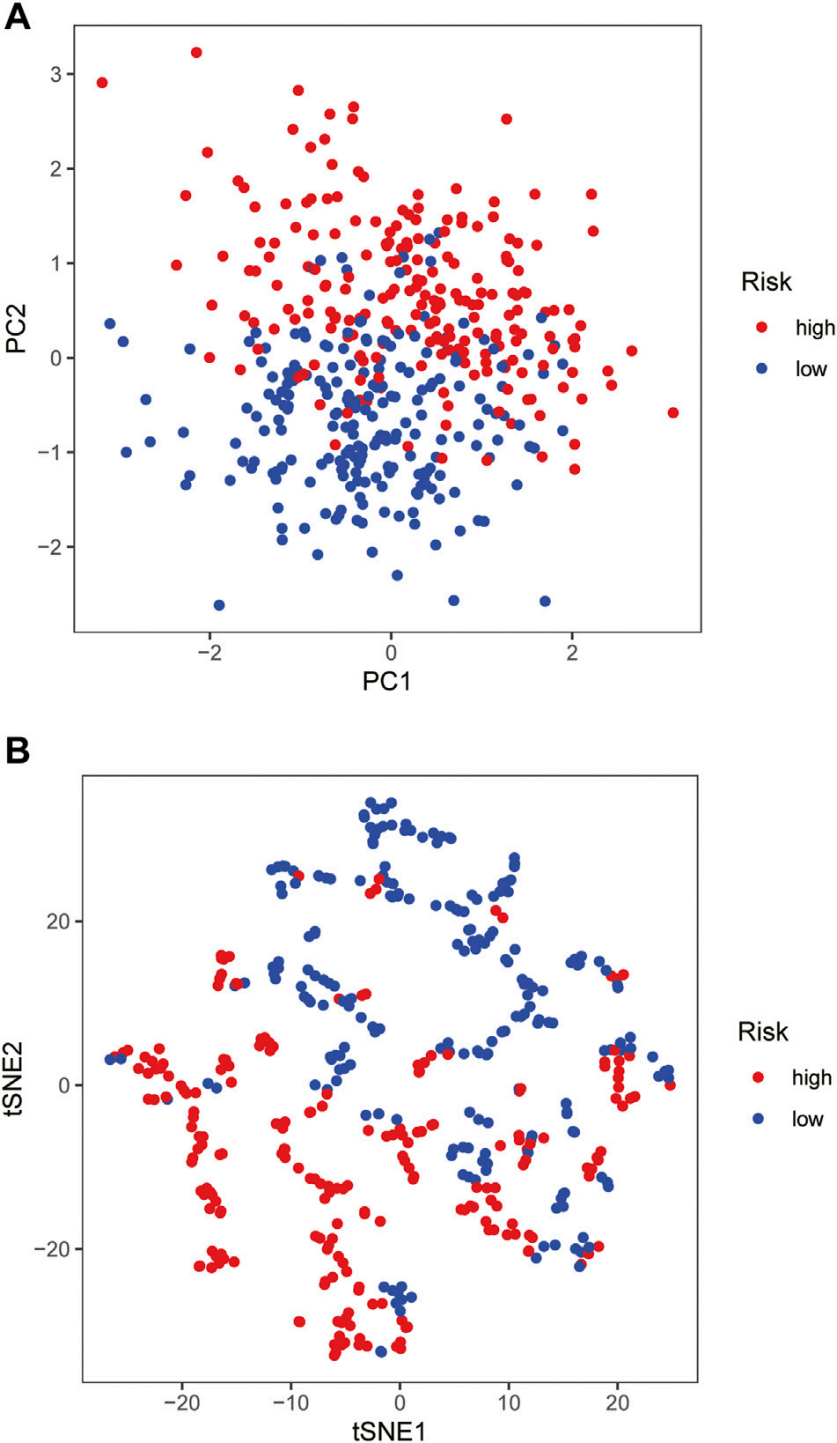
Construction of risk-scoring model **(A,B)** PCA and t-SNE based on the expression profile of the 3 selected signature genes separated different risk groups.

The samples screened from the TCGA database were randomly assigned to the training set for the construction of the model and the validation set for accuracy estimation according to the ratio of 1:1. The clinical features of all patients are shown in detail in Table 1. There were no statistically significant differences in clinical features between patients in the training set and validation set. Based on the training data set, the prognosis-related glycosylation genes were screened by univariate Cox regression (*p* < 0.05), including two low-risk genes [hazard ratio (HR) < 1] and one high-risk gene [risk ratio (HR) > 1]. Then, multivariate Cox analysis was performed to screen three genes related to the glycosylation with prognostic significance (*p* < 0.05), namely *BST2, IGHA2,* and *SLC35A2*. These three genes were used to construct a risk-scoring model. According to the risk score formula and the median risk score, the patients with breast cancer were divided into the high- and low-risk groups. The PCA and t-SNE showed that the high- and low-risk groups had different distribution directions, suggesting that the risk-scoring model could clearly divide BRCA patients into two groups (Figures 2A,B).

**TABLE 1:** The association between risk score and patients’ clinical features in the training set.

Variables	Training Set		Validation Set		*p*-Value
	(n = 432)		(n = 429)		
	No.	%	No.	%	
Age					0.986
≤65	319	73.8	318	74.1	
>65	113	26.2	111	25.9	
Stage					0.870
I	72	16.7	79	18.4	
II	254	58.8	251	58.5	
III	97	25.5	92	21.5	
IV	9	2	7	1.6	
T stage					0.622
T1	111	25.7	115	26.8	
T2	253	58.6	258	60.1	
T3	51	11.8	45	10.5	
T4	17	3.9	11	2.6	
N stage					0.504
N0	218	50.5	201	46.9	
N1	141	32.6	147	34.2	
N2	51	11.8	50	11.7	
N3	22	5.1	31	7.2	
M stage					0.812
M0	423	97.9	422	98.4	
M1	9	2.1	7	1.6	

### Evaluation and Validation of the Aberrant Glycosylation-Related Risk-Scoring Model

**FIGURE 3:**
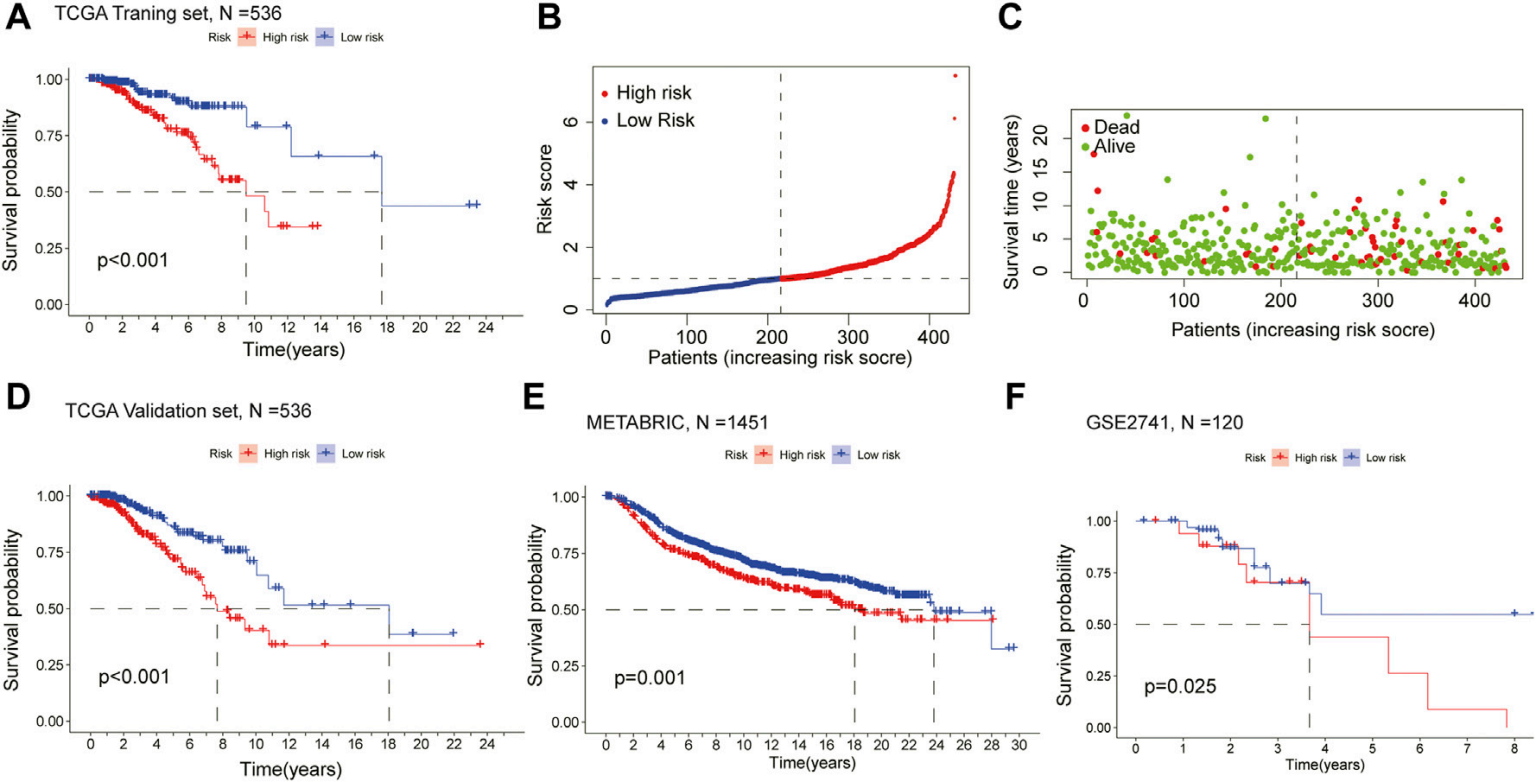
Assessment of the prognostic model in mRNA expression database **(A)** Survival curve of the high- and low-risk groups in the training set (*n* = 536) (log rank test, *p* < 0.05) **(B)** The distribution of the risk scores in the training set **(C)** The distributions of survival status, OS, and risk score in the training set **(D)** Kaplan-Meier survival curve of validation set (*n* = 536) comparing the high- and low-risk groups **(E)** Kaplan-Meier survival curve of the high- and low-risk groups in the independent test set METABRIC (*n* = 1451) **(F)** Kaplan-Meier survival curve analysis in the independent test set GSE2741 (*n* = 120) comparing two risk groups. All the K-M survival analyses use log-rank tests to determine significant differences between two groups, *p* < 0.05.

Kaplan-Meier survival analysis showed that the OS of the high-risk group was lower than that of the low-risk group in the training cohort (*p* < 0.001) (Figure 3A), demonstrating the excellent predictive value of the risk-scoring model in the training set. The risk curves showed the survival status and risk scores of each breast cancer sample, which was calculated and ranked based on the signature model (Figure 3B). The scatter plot represented the OS status of BRCA patients according to the risk score, suggesting that the higher the risk scores were, the higher the number of death was (Figure 3C). Similarly, with Kaplan-Meier survival analysis using the validation cohort and external independent test sets, the OS rate of the high-risk group was lower than that of the low-risk group (*p* < 0.05), confirming that the risk-scoring model had a robust prognostic value (Figures 3D–F).

### Explore the Independent Prognostic Factors of Breast Cancer

**FIGURE 4:**
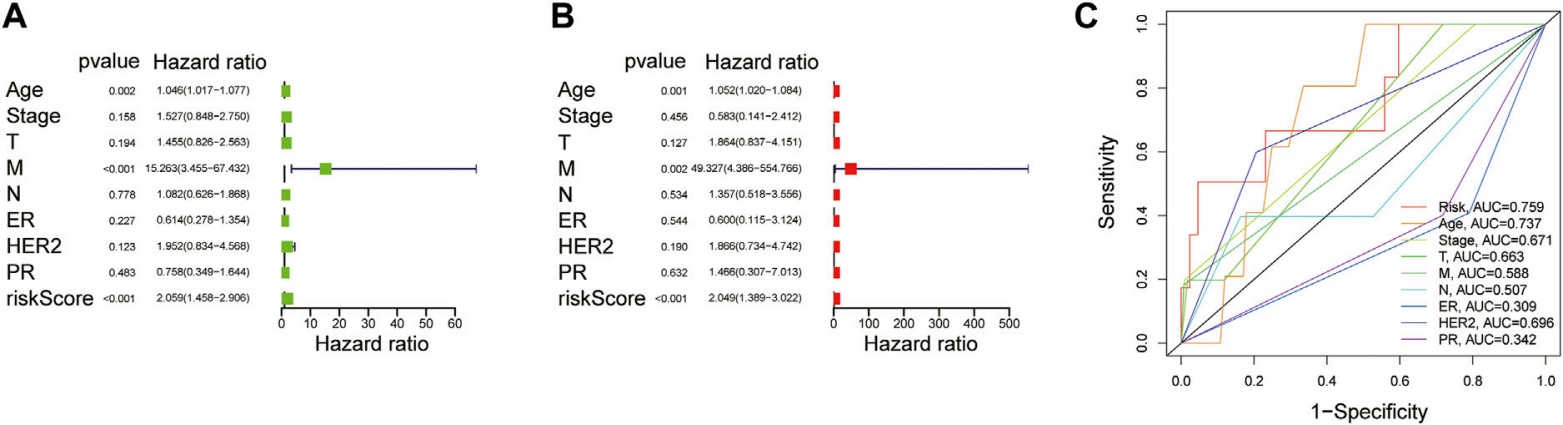
Explore the prognostic value of risk score and clinical features **(A)** Univariate Cox regression analysis of breast cancer patients **(B)** Multivariate Cox regression analysis of breast cancer patients (Wald test, *p* < 0.05) **(C)** The AUC for risk model scores and clinical features according to the ROC curves. Clinical features: Age, ER, PR, HER-2, TNM stage, T, N, and M.

To evaluate whether the established risk-scoring model is an independent prognostic factor for breast cancer, univariate and multivariate Cox regression was performed. The HR of the risk scores and 95% CI were 2.059 and 1.458–2.906 (*p* < 0.001), respectively, in univariate Cox regression analysis (Figure 4A), which were 2.049 and 1.389–3.022 (*p* < 0.001) in multivariate Cox regression analysis, respectively (Figure 4B). The result demonstrated that the risk score was a significant prognostic factor independent of multiple clinicopathological parameters such as the expression level of estrogen receptor (ER), progesterone receptor (PR), human epidermal growth factor receptor (HER-2) as well as the M stage, N stage, T stage, tumor stage. Additionally, compared with other clinicopathological factors, the AUC of the risk score for 1-year OS shown by ROC analysis reached 0.759, which was superior to other clinicopathological variables (Figure 4C). In summary, it can be concluded that the aberrant glycosylation-related risk scoring model is a significant independent prognostic factor for BRCA patients.

### Subgroup Analysis

**FIGURE 5:**
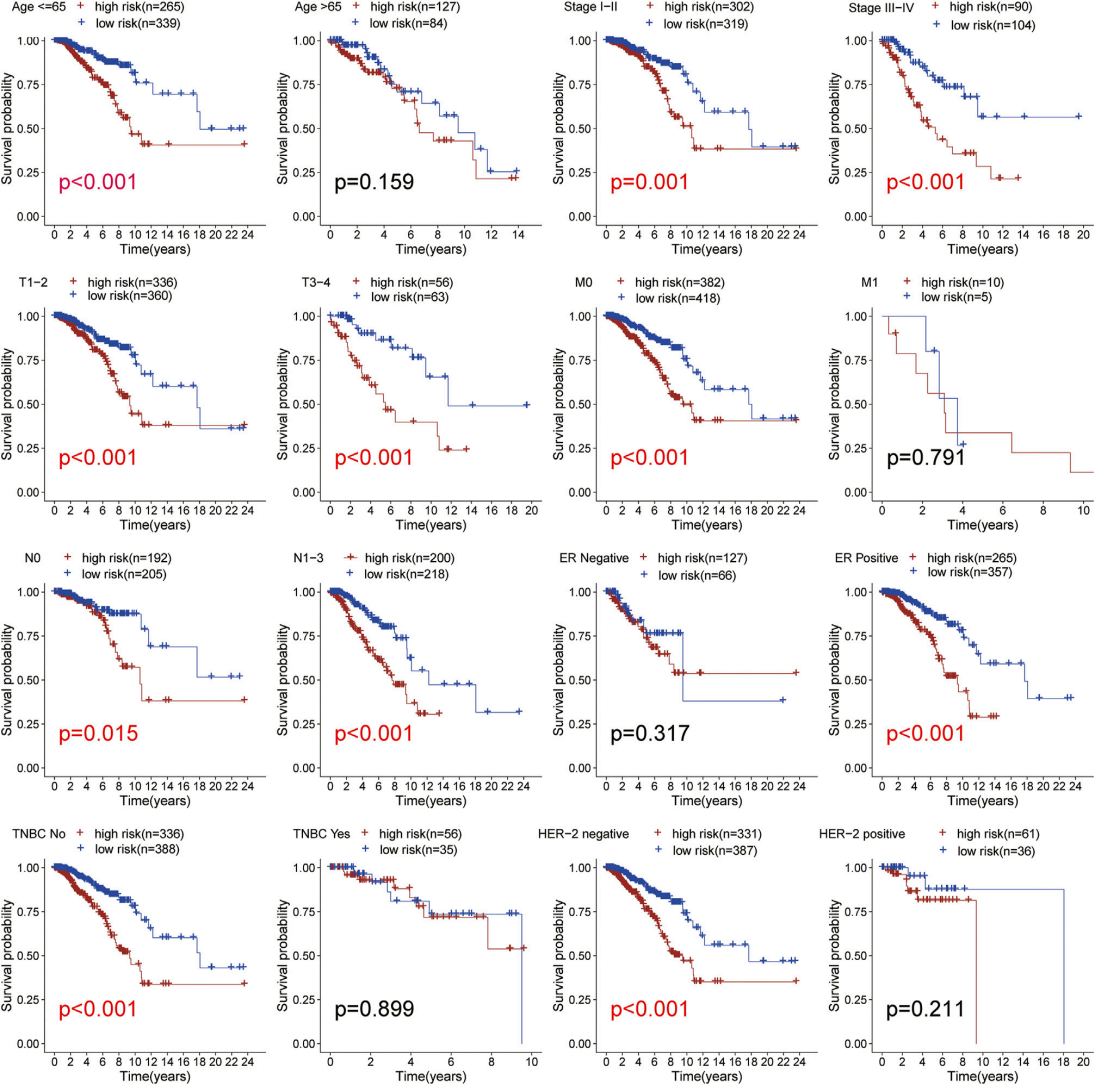
Subgroup analysis. Kaplan-Meier survival curve analysis of patients with high-vs low-risk scores in different subgroups including age, TNM stage, ER status, HER-2 status, TNBC status. All the K-M survival analyses use log-rank tests to determine significant differences in subgroups.

We used the TCGA BRCA samples to verify the relationship between the risk score and the prognosis of clinical features and emphasize the molecular heterogeneity of BRCA. After comparing a statistical difference in OS between the two risk groups with Kaplan Meier survival curve, the results showed that the OS of the high-risk group was lower than that of the low-risk group among age ≤65, ER positive, HER-2 negative, M0, N0, N1-N3, stage I-II, stage III-IV, T1-2, T3-4, and non-TNBC subgroups (*p* < 0.05; Figure 5). In addition, the low-risk group had higher OS in the TNBC and HER-2 positive subgroups from METABRIC (Supplementary Figure S1A,S2). The results suggested that the risk score is closely related to the clinical features of BRCA and can be used as an effective auxiliary tool to predict the BRCA prognosis.

### Clinical Evaluation Ability of the Risk-Scoring Model

**FIGURE 6:**
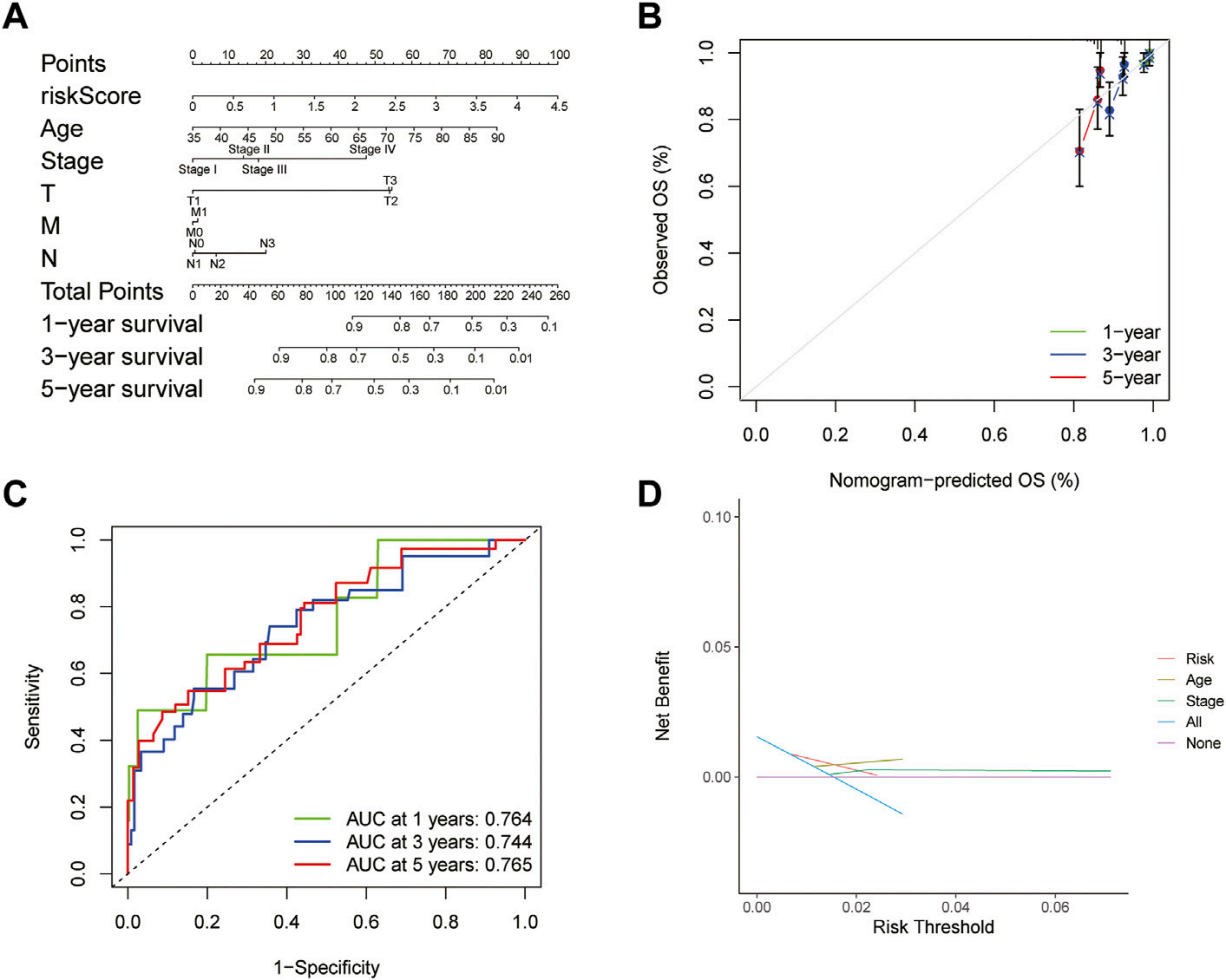
Construction and evaluation of nomogram **(A)** Nomogram for predicting the 1-, 3-, and 5-year survival rates based on the risk score **(B)** Calibration curve of the nomogram **(C)** ROC curve analysis evaluated the prediction performance of nomogram **(D)** Decision curves of “risk”, “age”, “stage”, “all” and “None” models.

A predictive nomogram based on the integration of risk score, pathological stage, and age integration was established in the TCGA cohort (Figure 6A). The C-index of the nomogram was 0.676 in the TCGA cohort, indicating that the nomogram had a good predictive performance. The calibration curve analysis verified that the nomogram was reliable and accurate, which demonstrated that the predictive probability of 1-, 3- and 5-year OS was ideally consistent with actual observation (Figure 6B). With the analysis of the ROC curve, the AUC value was 0.764, 0.744, and 0.765 for 1-, 3-, and 5-year OS, respectively, indicating that the risk-scoring model had excellent predictive accuracy (Figure 6C). In addition, DCA analysis was performed to evaluate the predictive value of the nomogram in clinical decision-making (Figure 6D). The above all indicated that the risk-scoring model and nomogram had high reliability.

### Gene Set and Function Enrichment Analysis

**FIGURE 7:**
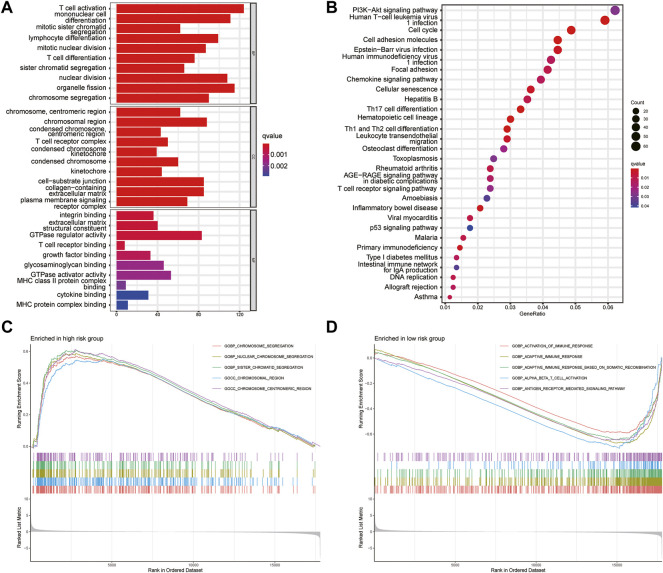
Gene set enrichment analysis **(A)** GO analysis the first 10 items about the enrichment of BP, CC and MF were shown in the bar chart **(B)** The top 30 terms of KEGG pathways enrichment were displayed in the bubble chart **(C,D)** Gene set and function enrichment analysis of differentially expressed genes between the high-risk group low-risk group. *p* < 0.05 and *FC* > 1.2 (or *FC* < 0.83) were set as the cut-off values.

To analyze the pathways related to the risk-scoring model, GO and KEGG enrichment analysis were performed. GO analysis showed that the differential genes between the high- and low-risk groups were enriched in T cell activation, T cell receptor binding, integrin binding, and nuclear division (Figure 7A). KEGG enrichment showed the differential genes between two groups related to PI3K-Akt signaling pathways, cell adhesion molecules, and leukocyte differentiation (Figure 7B). GSEA was further used to investigate the key signaling pathways in different risk groups (Figures 7C,D). The results revealed that chromosome segregation was enriched in the high-risk group while T cell activation, immune response, antigen receptor-mediated signaling pathway were enriched in the low-risk group. These findings explained the poor survival in the high-risk group and may help us gain insight into the implication of aberrant glycosylation-related signature.

### Tumor Immune Microenvironment of BRCA

**FIGURE 8:**
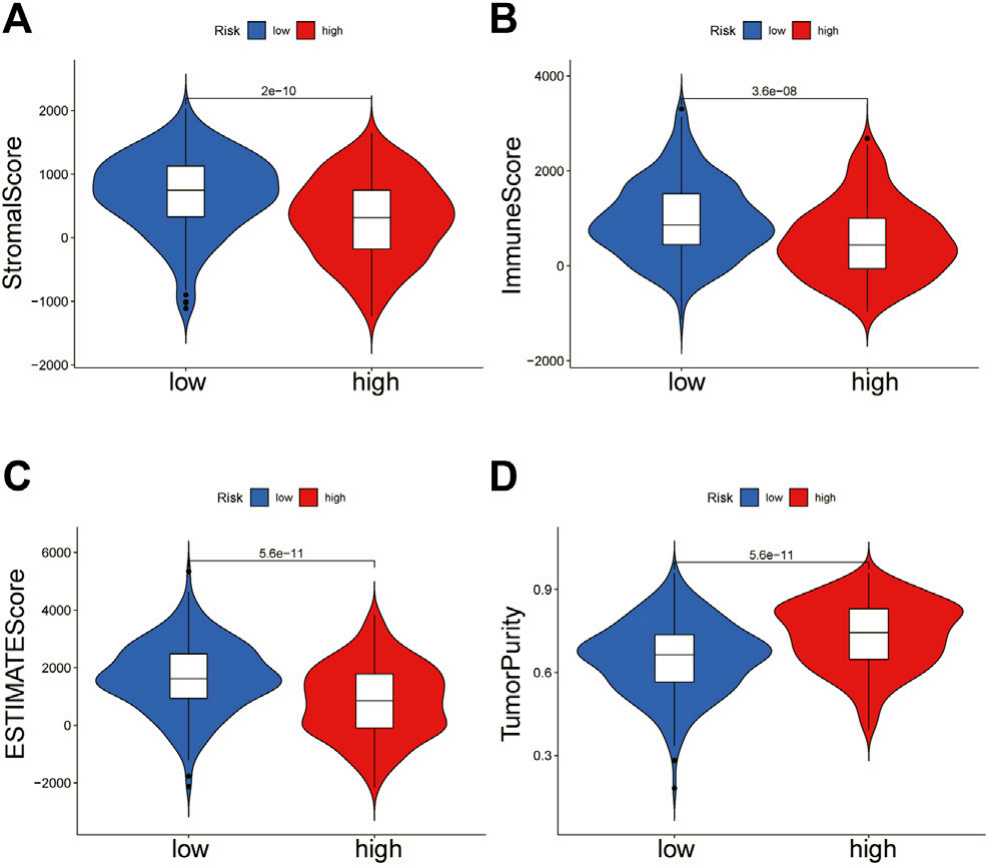
ESTIMATE algorithm calculates immune score. The violin chart showed the comparison of stromal scores **(A)**, immune scores **(B)**, ESTIMATE scores **(C)**, and tumor purity **(D)** between the high-risk group and the low-risk group (Wilcoxon test, *p* < 0.05).

**FIGURE 9:**
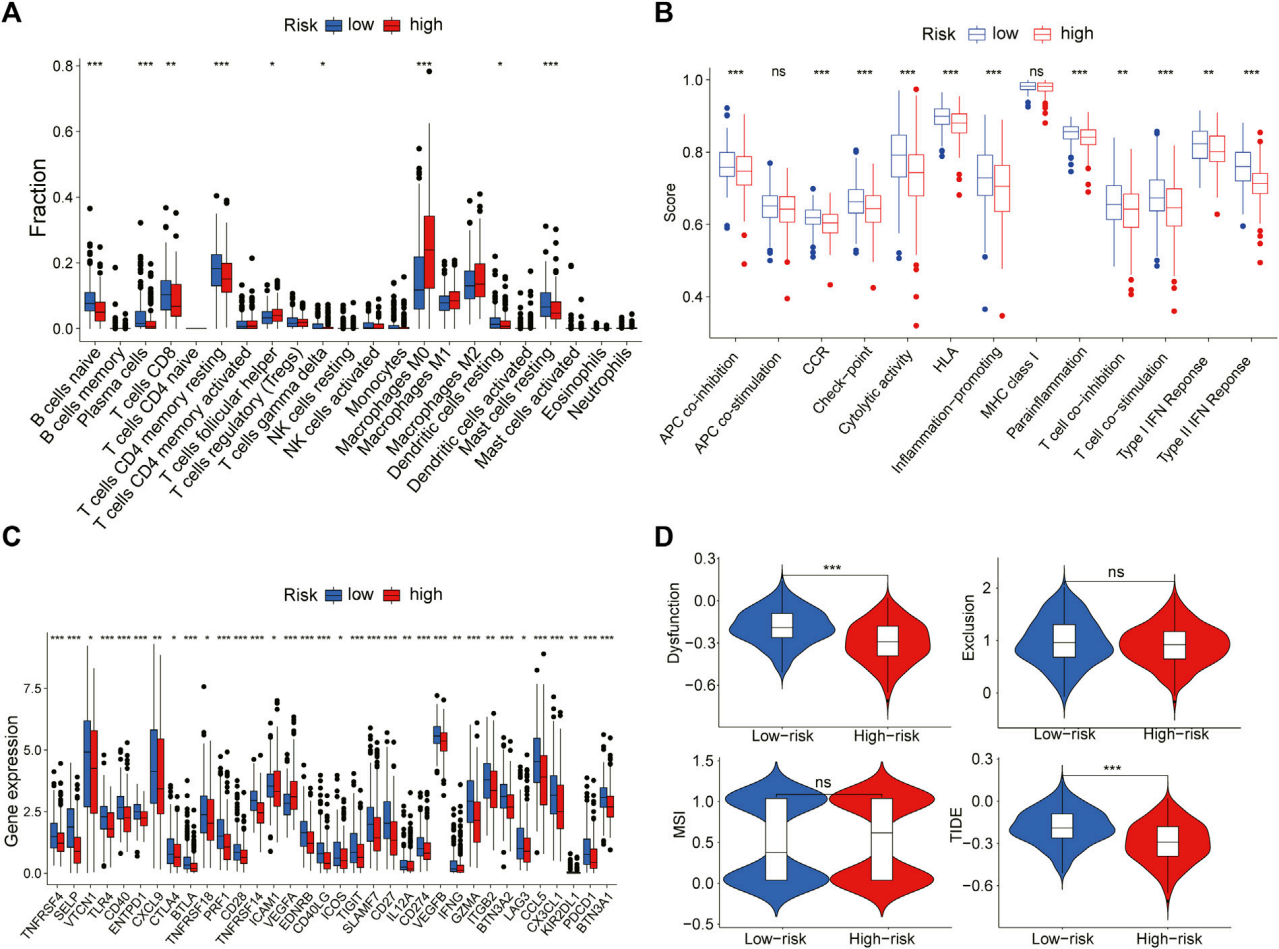
The relation between risk scoring model and the immune microenvironment **(A)** The plot showed the estimated proportion of 22 immune cell in the high- and low-risk group **(B)** Box plot of differences in immune related ssGSEA scores between the two groups **(C)** Box plot of differences in the expression levels of immune related including immune checkpoint genes between the two groups **(D)** The violin plot showed the difference in dysfunction, exclusion, MSI and TIDE signature between two risk groups (Wilcoxon test, *p < 0.05, **p < 0.01, ***p < 0.001, ns, no significant difference).

To further explain the difference in survival between the two groups, we explored the relationship between glycosylation and the tumor immune microenvironment. Stromal scores, immune scores, and ESTIMATE scores were evaluated by the ESTIMATE package, all of which were higher in the low-risk group (*p* < 0.001; Figures 8A–C). In contrast, patients in the high-risk group were associated with significantly higher tumor purity (*p* < 0.001) (Figure 8D). After the proportion of 22 immune cell types was calculated by CIBERSORT, there were significant differences in the infiltration scores of nine immune cells between the two groups (Figure 9A), including B cell naive, plasma cell, T cell CD8 +, T cell CD4 + memory resting, T cell gamma delta, Macrophage M0, Macrophage M2, mast cell resting, and T cell follicular helper (*p* < 0.05). Subsequently, we used Spearman Correlation Analysis to explore the relationship between risk score and immune cell infiltration. The results showed that the low-risk group had a higher infiltration level of B cells naive, mast cells resting, plasma cells, T cells CD4 memory resting, T cells CD8, and T cells gamma delta (*p* < 0.05), while higher macrophages M0, macrophages M2 and T cells follicular helper were found in the high-risk group (*p* < 0.05) (Supplementary Figure S3). The ssGSEA was performed to quantify the enrichment scores of 13 immune cell-related functions between the two risk groups. The results showed that the scores of APC co-inhibition, CCR, Check-point, cytolytic activity, HLA, Inflammation-promoting, Para inflammation, T cells co-stimulation, Type I IFN response, Type II IFN response of patients in the low-risk group were higher (*p* < 0.01, Figure 9B), indicating that the low-risk group had higher immune infiltration than the high-risk group did. We further explored the immune checkpoints, and the result showed that the distribution of immune checkpoint-related molecule expression was significantly different between the high- and low-risk groups. The difference analysis confirmed that the expression of 22 immune promoting checkpoints (TNFRSF4, SELP, TLR4, CD40, ENTPD1, CXCL9, TNFRSF18, PRF1, CD28, TNFRSF14, ICAM1, CD40LG, ICOS, CD27, IL12A, IFNG, GZMA, ITGB2, BTN3A2, CCL5, CX3CL1, BTN3A1) in the low-risk group was higher, indicating that the prognosis of the low-risk group was better than that of the high-risk group (*p* < 0.05) (Figure 9C), that may provide the potential targets of immunotherapy for BRCA patients. In conclusion, the low-risk group had higher Tumor infiltrating lymphocytes (TILs), of which the prognosis was better, compared with the high-risk group.

### Genomic Mutation Analysis

**FIGURE 10:**
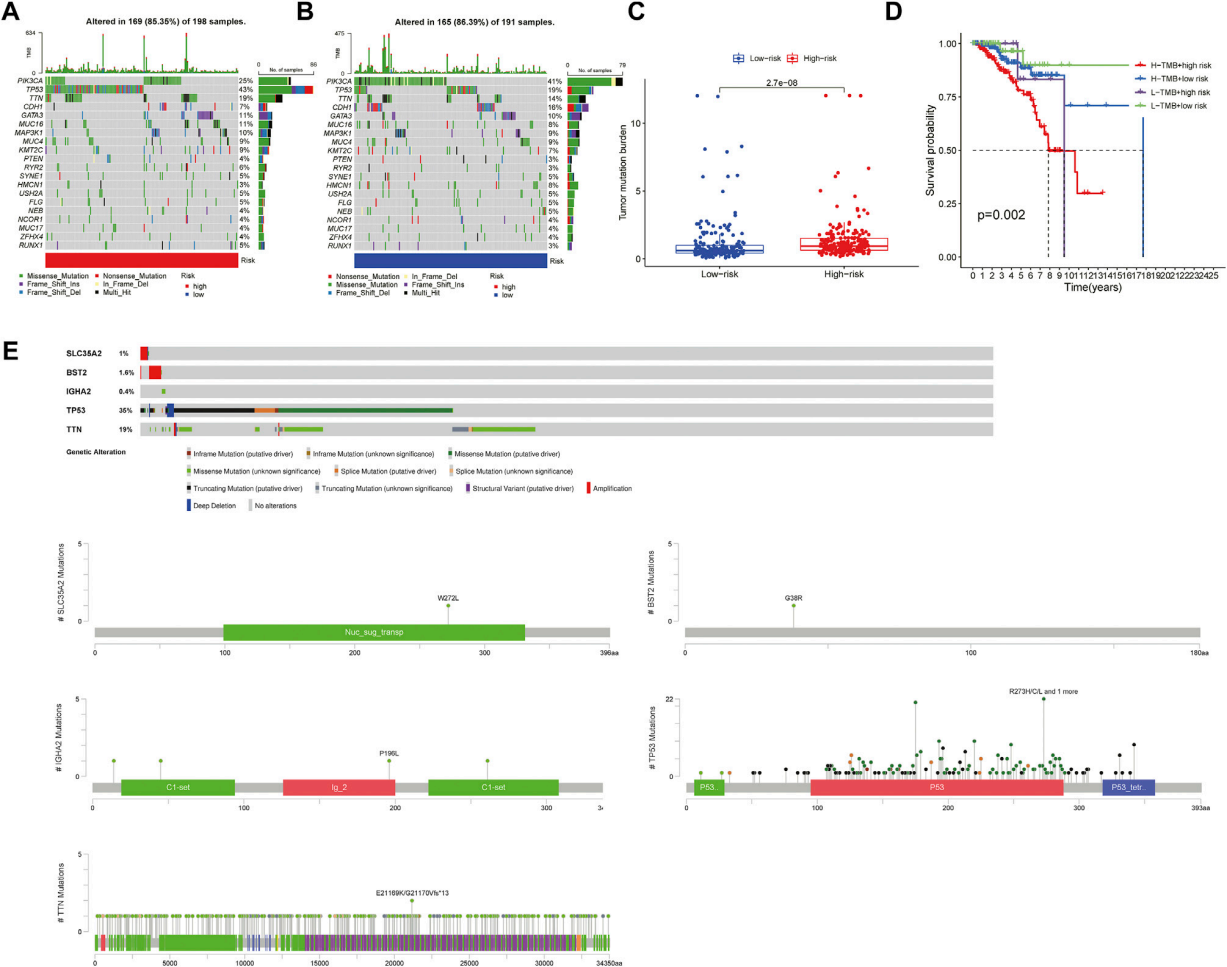
Mutation analysis in the high-risk group and the low-risk group **(A,B)**. The waterfall chart showed the mutation frequency of the high- and low-risk groups **(C)** The box plot reflected the correlation between the risk score and the TMB scores (Wilcoxon test, *p* < 0.05) **(D)** The Survival curve showed the joint effect of TMB score and risk score on the prognosis of patients (log rank test, *p* < 0.05) **(E)** The plot showed the mutation type of key genes and functional domains where the mutations were enriched.

Through the downloaded somatic mutation data, the mutation frequency of the high-risk group and the low-risk group was calculated, and the waterfall chart was drawn to confirm the difference in the distribution of somatic mutations. It found that 169 of 198 (85.35%) BRCA samples in the high-risk group and 165 of 191 (86.39%) BRCA samples in the low-risk group displayed genetic mutations, and missense mutation was the most common variant classification. Moreover, in the high-risk group, PIK3CA had high genetic alterations (25%), which was just junior to the genetic alterations of TP53 (43%). In the low-risk group, PIK3CA had the most genetic alterations (41%) (Figures 10A,B). We also found that the TMB of patients in the high-risk group was significantly higher than that in the low-risk group (*p* < 0.001, Figure 10C), indicating that BRCA patients in the high-risk groups may derive good outcomes from immune checkpoint inhibitor treatments. The combined survival analysis showed that the prognosis of low-risk and low TMB patients was significantly better than that of high-risk and high TMB patients (*p* < 0.01, Figure 10D). Mutations of the genes for constructing the risk-scoring model and the genes with higher mutation frequency in the high-risk group showed that mutations in *SLC35A2* and *BST2* were mainly related to gene amplification, while mutations in *IGHA2*, *TP53*, and *TTN* were mainly related to missense mutations. By the CBIOPORTAL database, the domain where the mutations were located was defined, including *SLC35A2* mutations were localized in the nucleotide sugar transporter domain, *IGHA2* mutations were enriched in the immunoglobulin domain, the majority of *TP53* mutations were centralized in the *P53* DNA binding domain, and *TTN* mutations were localized in the immunoglobulin I-set domain, fibronectin type III domain, protein kinase domain, titin Z, and PPAK motif (Figure 10E).

### Analysis of Immunotherapy Response and Drug Sensitivity

**FIGURE 11:**
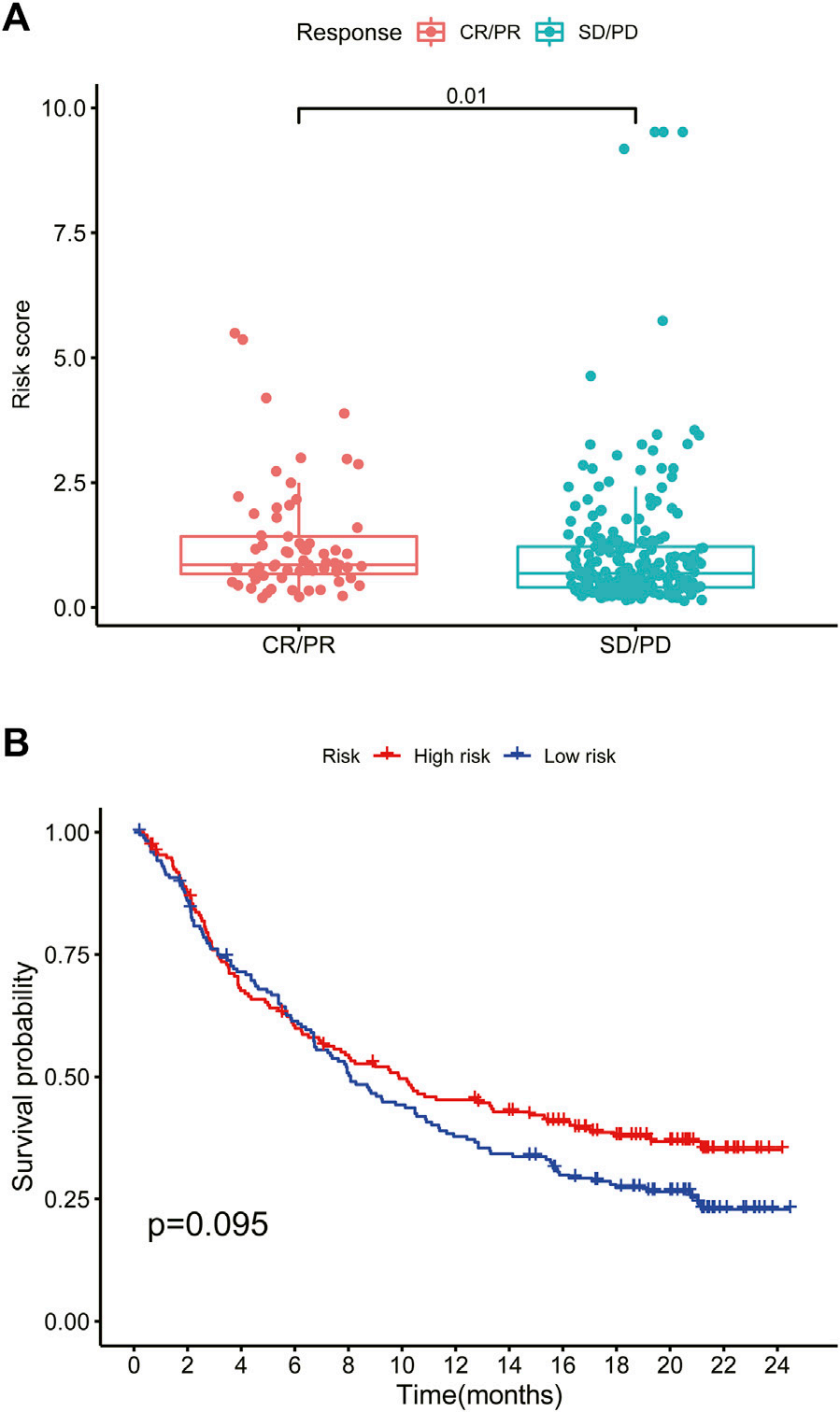
High-risk scores predict immunotherapy response **(A)** Box plot of the effect of the high- and low-risk groups after immunotherapy (Wilcoxon test, *p* < 0.05) **(B)** Survival curve of the high- and low-risk groups after immunotherapy. CR, complete response. PR, partial response. SD, stable disease. PD, progressive disease (log rank test, *p* < 0.05).

**FIGURE 12:**
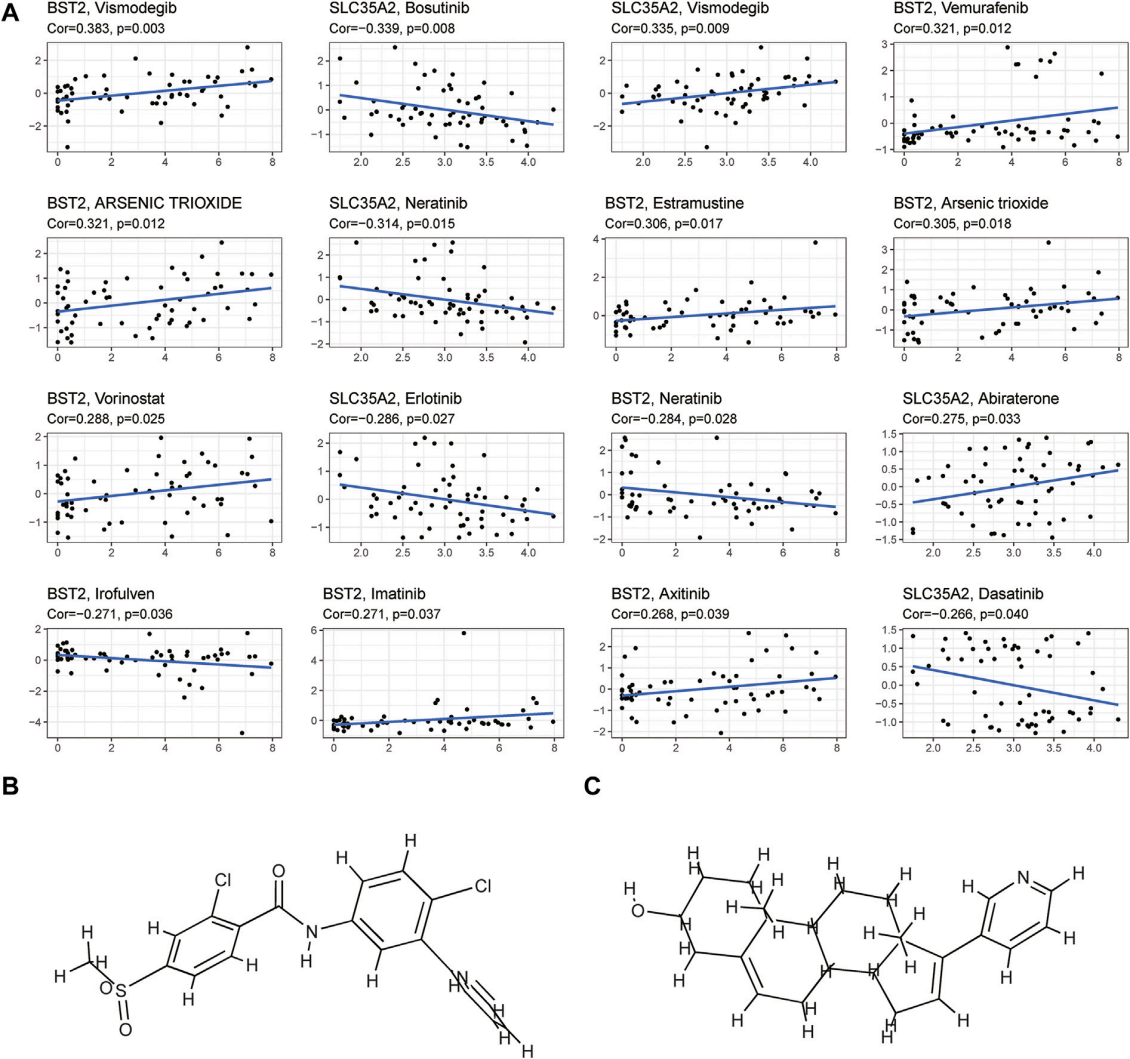
Drug sensitivity analysis **(A)** The plot showed the correlation between the expression levels of key genes in the model and cell sensitivities to certain drugs (*p* < 0.05) **(B,C)** The plot showed the three-dimensional structure of two drugs (Vismodegib and Abiraterone) whose sensitivities were positively correlated with the expression of *SLC35A2*.

We explored the OS difference after immunotherapy between the high- and low-risk group in the phase II trial, in which atezolizumab was treated with platinum-treated locally advanced or metastatic urothelial carcinoma (IMvigor210). The patients in the high-risk group had a better effect in receiving immunotherapy (*p* < 0.05, Figure 11A). Although there was no significant statistical difference, it was found that the prognosis of the high-risk group was better than that of the low-risk group after immunotherapy (*p* = 0.095, Figure 11B). The better response to immunotherapy in the high-risk group may be due to the relatively higher TMB that we had demonstrated before. A similar correlation between TMB and our risk scoring model was identified in TCGA BLCA (Supplementary Figure S4). Then, TIDE was further used to assess the potential immunotherapy effect in the high- and low-risk groups. The high-risk group had a lower TIDE score, which represented a lower possibility of immune escape, suggesting the BRCA patients in the high-risk group could benefit more from immune checkpoint inhibitor therapy (Figure 9D). Besides, the low-risk group got a higher T-cell dysfunction score (Figure 9D). Through further analyzing the drug sensitivity by comparing the expression levels of model genes and drug response data from CELLMINER, we obtained the drugs with the most statistical significance (Figure 12A). The expression of *SLC35A2* was positively correlated with the sensitivity of two drugs (Vismodegib and Abiraterone) (HR > 1, *p* < 0.05), in turn, more sensitive in the high-risk group than in the low-risk group. The three-dimensional structures of the two drugs were obtained from the PubChem database, which provided potential guidance for chemotherapy in high-risk BRCA patients (Figures 12B,C).

## Discussion

Breast cancer is the most common cancer in women, accounting for nearly 25% of all cancer cases in women. It is also the leading cause of cancer deaths among elderly women (Hadadi et al., 2020; Siegel et al., 2021). The 5-year survival rate for patients with metastatic or stage IV breast cancer is 22% (www.cancer.org). The disease has heterogeneity and possesses a diverse mutational landscape, suggesting differences in patients’ response to treatments and lack of targeted treatment for patients in specific breast cancer subtypes, which indicates the need to improve the guidance for treatment strategies. In recent years, with the progress of high-throughput sequencing and data analysis, it has become a vital biomedical research tool, which can be used for prognosis prediction, recurrence monitoring, and clinical stratification (Z. Wang et al., 2009b; I. D. Kyrochristos et al., 2019). Therefore, it is urgent to apply this tool to prevent and treat breast cancer. Many shreds of evidence had shown that aberrant glycosylation had multiple effects on cancer’s occurrence, progression, invasion, and metastasis (T. D. Rao et al., 2017; J. Cui et al., 2018). Potapenko et al. reported not only that there are significant differences in the expression characteristics of glycosylation-related genes in breast cancer compared to normal breast tissue, but also that glycosylation -related genes show significant differences in expression between breast cancer subtypes and may be associated with patient prognosis and suggested that alterations in glycosylation pathways may occur at different time points in the carcinogenesis process (I. O. Potapenko et al., 2010; I. O. Potapenko et al., 2015). At present, there was still no prognostic model based on glycosylation-related genes for breast cancer. Given the critical impact of glycosylation on tumors, we had developed a prognostic model based on three glycosylation-related genes (*BST2*, *IGHA2, SLC35A2*).

Evidence showed that *SLC35A2* belonged to the solute carrier family *SLC35* of human nucleoside sugar transporters, and encoded an X-linked transporter that transports uridine diphosphate—galactose from the cytoplasm to the lumen of the Golgi apparatus and endoplasmic reticulum. Pathogenic variation was associated with congenital glycosylation disorder characterized by epileptic encephalopathy (D. Quelhas et al., 2021). However, rare research had shown its relationship with cancer. Therefore, exploring the mechanism of *SLC35A2* is in high demand. In our study, the established prognostic model showed that the OS rate of patients in the high-risk group was significantly lower than that in the low-risk group through Kaplan Meier survival analysis (*p* < 0.001). Then, we testified the reliability of the model by using the validation set and external independent test sets. By comparing the risk score with other clinicopathological factors with a ROC curve, it was found that the risk model had a higher prognostic value than other clinicopathological factors. We also constructed a nomogram based on the age, TMN stage, and risk score to improve the accuracy of clinical decision-making. The risk score and nomogram had high reliability by calculating C-index and decision curve analysis. Subsequently, we divided the samples into multiple groups to demonstrate the application of the model in specific categories. The risk model had an excellent predictive value among age ≤65, ER positive, HER-2 negative, M0, N0, N1-N3, Stage I-II, Stage III-IV, T1-2, T3-4, non-TNBC subgroups (*p* < 0.05). In M1 and HER-2 positive subgroups, although there was no significant difference between the high- and low-risk groups, which might be ascribed to the small number of samples and the short follow-up time of patients in the subgroups, the survival time of the low-risk group was higher than that of the high-risk group. There was no difference in the ER negative and TNBC subgroups, resulting from the small sample size. We successfully validated the predictive value of the model in HER-2 positive and TNBC subgroups by METABRIC. Regarding the immunobiology of BRCA, TILs as an important biomarker in predicting the efficacy and outcome of treatment were worth exploring in depth. In breast cancer patients, loss of the anti-HER-2 CD4^+^ Th1 immune response is independently correlated with disease recurrence (J. Datta et al., 2016). CD8^+^ TILs, Th1 CD4^+^ TILs can influence anti-tumor immune response in breast cancer (A. Basu et al., 2019). Several clinical trials have also shown an increased pathological complete response associated with a high density of TILs (Y. Issa-Nummer et al., 2013; C. Denkert et al., 2015). We explored the relationship between model grouping and scores of immune infiltrating cells. The results showed that the low-risk group had a higher infiltrative proportion of B cells naive, Mast cells resting, Plasma cells, T cells CD4 memory resting, T cells CD8, T cells gamma delta, which was in keeping with the previous investigations, suggesting a correlation between glycosylation and TILs. Therefore, impaired anti-tumor immune function may account for the poor prognosis in high-risk patients. Subsequently, we explored the differences in somatic mutations between the high- and the low-risk groups. The results showed that the TMB of the high-risk group was higher, indicating that patients in this group were more likely to receive the benefits of immune checkpoint inhibitor treatment (Steuer and Ramalingam, 2018). Moreover, the high-risk group had a lower TIDE score, which represented a lower possibility of immune escape, suggesting the BRCA patients in the high-risk group could benefit more from immune checkpoint inhibitor therapy (P. Jiang et al., 2018). The combined survival analysis showed that the prognosis of low-risk and low TMB patients was significantly better than that of high-risk and high TMB patients (*p* < 0.01). Then we analyzed chemotherapy based on the key genes. Two drugs (Vismodegib, Abiraterone) were expected to be validated and applied for the high-risk breast cancer patients.

This study also has some limitations. First, as it is a retrospective study derived from public data, it lacks some information such as recurrence time and treatment records. Second, clinical trials are urgently needed to confirm whether inducing glycosylation could improve the efficacy of immunotherapy in human BRCA patients. Furthermore, the specific molecular mechanism of the gene in the risk-scoring model has not been fully explored.

## Conclusion

In conclusion, a novel prognostic model integrating glycosylation-related genes was firstly constructed. Through verification in the validation set and external independent test sets, the risk-scoring model has been proved to be an independent prediction model for predicting the prognosis of patients, which was correlated with immunotherapy effect and drug sensitivity. Moreover, we established a prognostic nomogram to predict the OS of patients with BRCA. The novel model might provide insights for predicting the prognosis of BRCA patients and suggestions to guide individual therapeutic strategies.

## Data Availability

Publicly available datasets were analyzed in this study. This data can be found here: The data could be downloaded at (https://www.cancer.gov; http://www.ncbi.nlm.nih.gov/geo; http://www.cbioportal.org; https://www.genecards.org)and the code used during the current study are available from the corresponding author on reasonable request.
