# Supplementary figures and images for: A Novel Signature Integrated of Immunoglobulin, Glycosylation and Anti-Viral Genes to Predict Prognosis for Breast Cancer
Source: Front Genet. 2022 Apr 1;13:834731. doi: 10.3389/fgene.2022.834731 (PMC9011196; doi:10.3389/fgene.2022.834731)

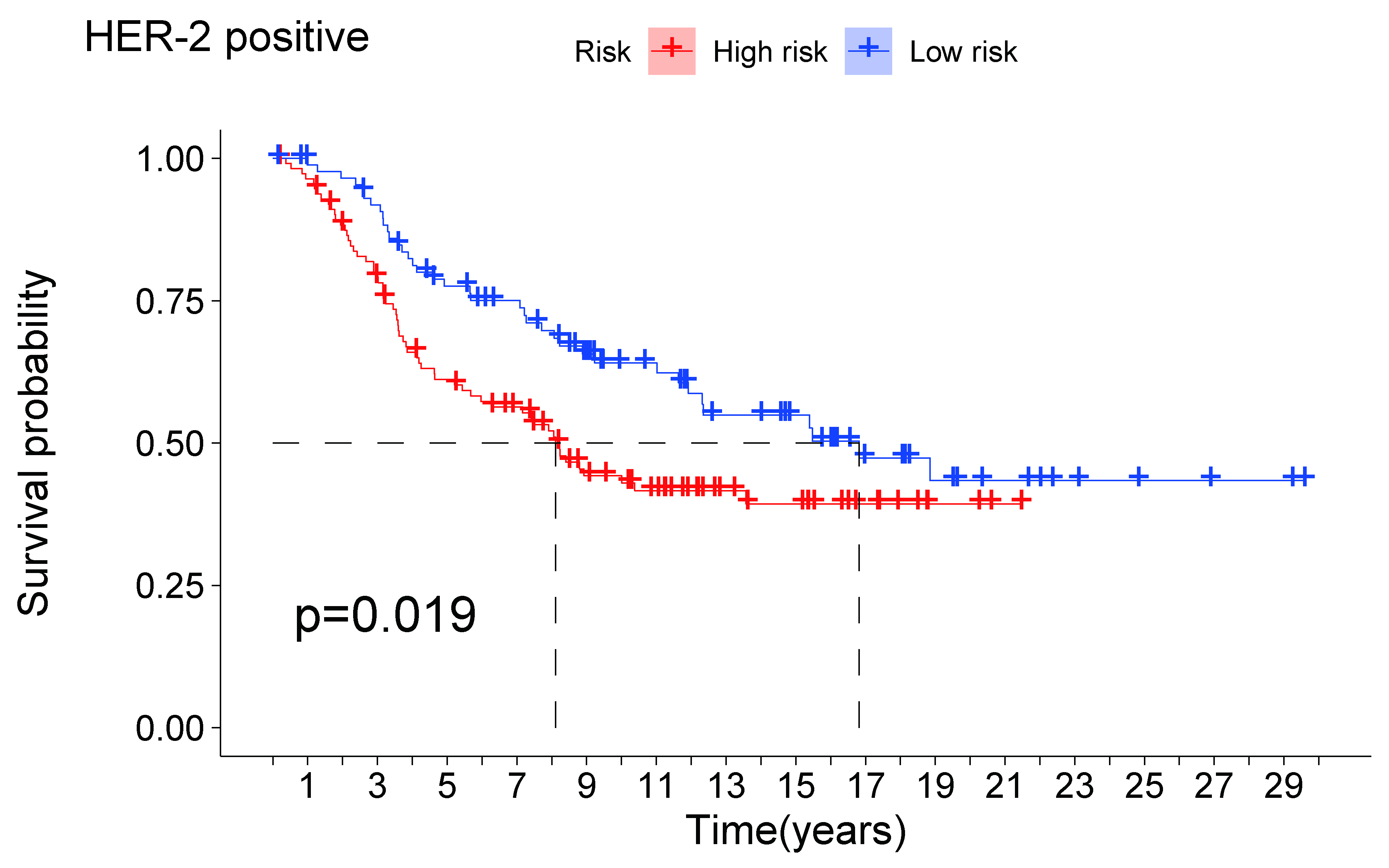

Supplement: Supplementary file 3 [file Image2.TIF]
